# Strategies for Managing the COVID-19 Pandemic and Lessons Learnt: An Irish Perspective

**DOI:** 10.3389/phrs.2025.1607427

**Published:** 2025-04-29

**Authors:** Alison Connolly, Peter Noone, Conor Buggy, Edel Costello, Frances Wright, Claire Farrell, Geraldine Lenehan, Michael Gillen, Peter Coulahan, Patrick Wall, Patricia McDonnell, Nuala Flavin

**Affiliations:** ^1^ UCD Centre for Safety and Health at Work, School of Public Health, Physiotherapy, and Sports Science, University College Dublin, Dublin, Ireland; ^2^ Regional Occupational Health Service, HSE Dublin North East, Naas, Ireland; ^3^ Department of Environmental Science, Faculty of Science, Atlantic Technological University Sligo, Sligo, Ireland; ^4^ Wright Environmental Services, Dublin, Ireland; ^5^ Occupational Hygiene Society of Ireland, Meath, Ireland; ^6^ BioPharmaChem Ireland, Ibec Head Office, Dublin, Ireland; ^7^ UCD Risk Management Office, University College Dublin, Dublin, Ireland; ^8^ UCD-Centre for Food Safety, School of Public Health, Physiotherapy and Sports Science, University College Dublin, Dublin, Ireland; ^9^ Alkermes Pharmaceutical Ireland Ltd., Dublin, Ireland

**Keywords:** COVID-19, pandemic, disaster management, occupational hygiene, health and safety

## Abstract

**Background:**

The World Health Organization (WHO) announced the end of the emergency phase of Corona Virus Disease 2019 (COVID-19) in May 2023. Nations across the world address the effects of the pandemic and the need to plan for future pandemics. Ireland heavily focused on isolation and social distancing to curb the infection rate early in the pandemic. These long and extended lockdowns gave Ireland a very unique experience during the COVID-19 pandemic.

**Analysis:**

Ireland’s COVID-19 pandemic management was discussed by an expert panel on the strengths in our national pandemic action plans, areas not sufficiently addressed and requirements to ensure future pandemic preparedness plans are robust.

**Policy Options:**

Recommendations include having a more strategic plan to protect society’s most vulnerable people, a flexible national policy that swiftly implements advances in scientific knowledge and good practices, a robust communication plan including localised information to prevent “pandemic fatigue,” and address challenges from restrictions, lockdowns and isolation such as mental health and wellbeing.

**Conclusion:**

Lessons learnt from the Irish COVID-19 pandemic experience can be utilised for pandemic preparedness plans, nationally and internationally.

## Background

A severe acute respiratory syndrome coronavirus 2 (SARS-CoV-2) was first identified in Wuhan, China, and was known as Corona Virus Disease 2019 (COVID-19). By early 2020, it was a worldwide pandemic [1]. Early evaluations identified countries worldwide as insufficiently prepared [2]. Many governments attempted to manage COVID-19 with repeated lockdowns, and many countries experienced high excess morbidity and mortality and poor economic recovery.

The end of the emergency phase of COVID-19 was in May 2023, but in reality, most countries have long relaxed most of their restrictions. To date, over 760 million cases and 6.9 million deaths have been recorded worldwide due to COVID-19, though the actual number is thought to be higher [1]. Furthermore, ongoing issues will occur for the foreseeable future, including the care for people with COVID-19 [3], constant emergence of variants, long-COVID [4], and mental health and wellbeing issues [5–8]. WHO Europe’s director statement “COVID-19 is here to stay” [9] emphasised the need for continued investment in vaccination campaigns and mitigation strategies [3].

Worldwide, countries must invest more in their national emergency/disaster preparedness and recovery plans [2]. Issues such as delayed detection of and response to the virus led to overburdening of our healthcare institutions [10]. Vaccinations and other mitigation controls (i.e., masking, social distancing) curbed the spread of the virus [11]. Despite the incredible speed of developing effective vaccines, the emergence of variants caused global public health efforts for several years [3].

Prior to the COVID-19 pandemic, healthcare expenditures in Ireland were 12% of modified Gross National Income. The healthcare service struggled to meet national needs with hospital bed occupancy at approximately 90%, 15% above the European average [12]. The number of practising doctors was 3.1 per 10,000, which is relatively low compared to international standards. There were 2.9 hospital beds per 10,000 inhabitants, with only 5.5 ICU beds per 10,000 people [12, 13]. At this time, over half a million patients were awaiting initial outpatient hospital appointments and many of the healthcare services were paused, postponed, or significantly altered when the COVID-19 restrictions were initiated; thus, deficits existed prior to the COVID-19 pandemic [12].

In Ireland, COVID-19 was first notified (HPSC Report) in March 2020, followed by over 1,000 cases notified within 3 weeks. Emergency legislation was established to regulate mass gatherings, lockdown designated areas, and travel restrictions. The first national lockdown was on 27 March 2020, with a ‘Stay at Home’ restriction and by September 2020, the Resilience and Recovery Plan for Living with COVID-19 was announced with a 5-level approach. The plan outlined restrictions for social gatherings, events, businesses, work, travel and within healthcare facilities (i.e., highest restrictions at Level 5). This plan also included information on testing and tracing with a dedicated workforce for COVID-19 testing and vaccination of at-risk groups and staff recruitment to assist with public health emergencies and PPE distribution among hospitals and the resuming of medical screening services and investment in E-health [14]. In December 2020, the vaccination programme was initiated and the National Immunisation Advisory Committee provided guidelines for prioritisation, vaccine types and recommendations (NIAC Immunisation Guidelines). Irish guidelines and guidance on best practices for triage, care and interventions were aligned with international guidance (i.e., WHO/ECDC) [14]. The Irish government activated the National Public Health Emergency Team (NPHET) who assisted with information transparency, and four official online databases were made freely available (HPSC; Department of Health; Open Data Portal; HSE). Ireland went through five distinct waves of the pandemic.

To date, Ireland has had nearly 10,000 deaths and over 1.75 million as per the WHO COVID-19 Dashboard [1] reported cases with a population of approximately 5 million people. Ireland imposed blanket restrictions through “lockdowns” to protect the vulnerable and not overwhelm hospitals. Ireland had some of Europe’s strictest and longest lockdowns, reducing the spread of the virus [13]. However, adverse effects from these measures also occurred from isolation, including mental health issues [5–8].

Ireland had a unique pandemic response (i.e., long and extended national lockdowns). The valuable perspectives and lessons learnt highlight important aspects that must be considered for future national preparedness plans internationally.

### Panel Discussion Format and Experts

**FIGURE 1:**
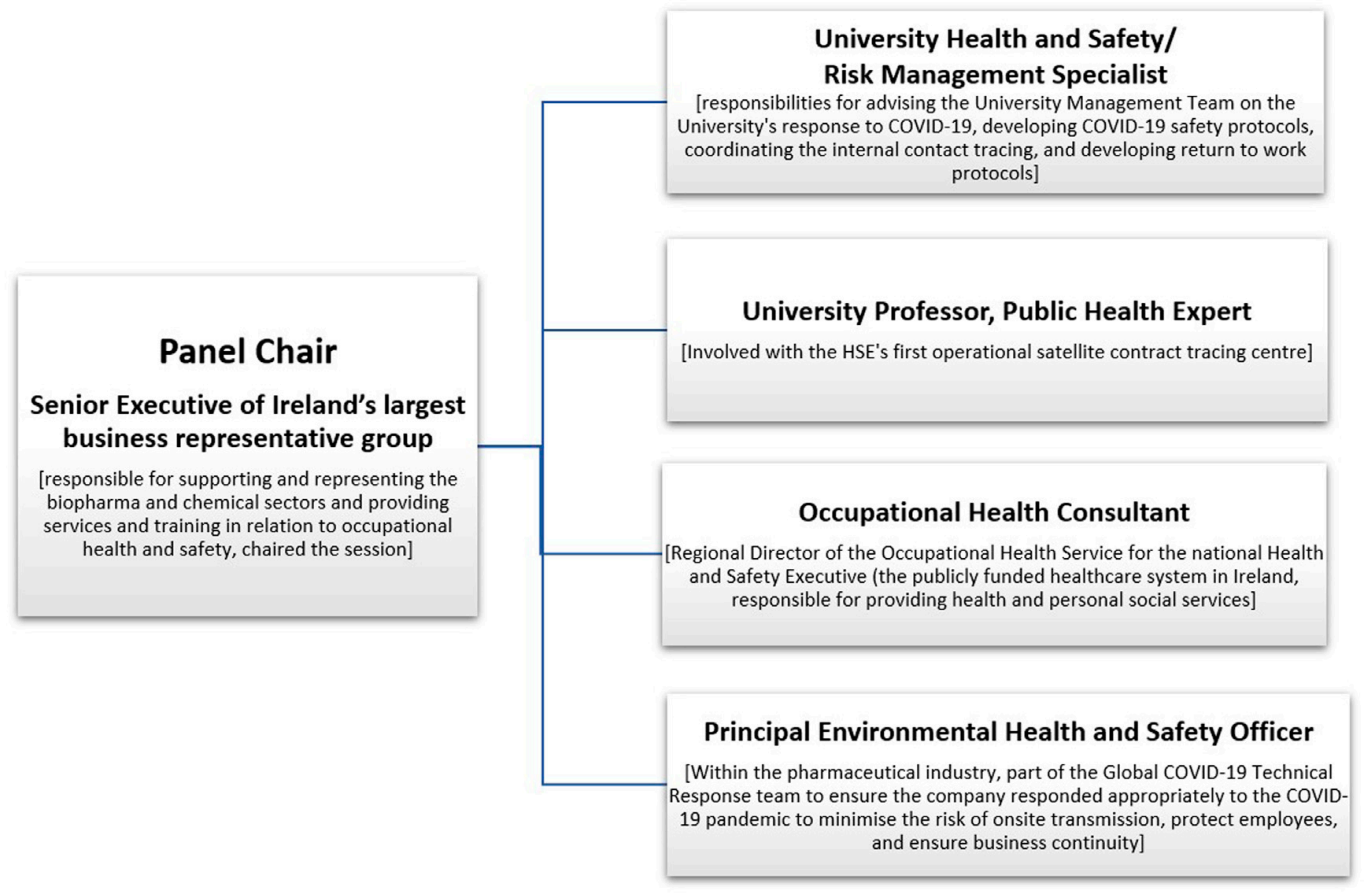
An overview of the panel format at the Occupational Hygiene Society of Ireland (OHSI) conference 2022: https://www.ohireland.org/ (Dublin, Ireland; 2022), including the panel members’ job titles and descriptions of their roles during the COVID-19 Pandemic. The panel chair moderated the session, and the following panel members were invited due to their expertise and roles during the COVID-19 Pandemic.

The Occupational Hygiene Society of Ireland (OHSI) 2022 annual conference held a panel discussion on “Lessons Learned from the Pandemic”. The panel discussion included national experts who represented key stakeholders who played a decisive role during the pandemic. A Senior Executive from Ireland’s largest business representative group chaired the session and national experts included professors, medical doctors, occupational hygienists, and Health and Safety (H&S) specialists from public bodies and private industry (Figure 1).

**FIGURE 2:**
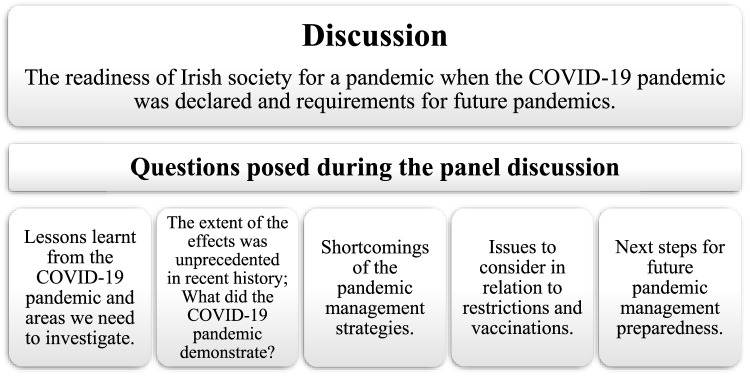
The theme of the panel discussion and the questions posed to the experts in relation to the COVID-19 pandemic which had five distinct pandemic waves from spring/early summer 2020 (2 March – 1 August 2020), late summer/autumn 2020 (2 August – 21 November 2020), winter 2020 to spring 2021 (22 November 2020–26th June 2021), the remainder of 2021 (27th of June 2021–18th December 2021) and onwards (19th December 2021 – onwards). There are currently no COVID-19 restrictions in Ireland outside of healthcare settings (Dublin, Ireland; 2022).

The chairperson posed questions about the pandemic response, the experience gained, and future improvements (Figure 2). Over 60 people attended the conference. Most event attendees are occupational hygienists within private, public, and regulatory agencies.

## Analysis

### Expert Panel Discussion

The cohorts most significantly affected in Ireland, with the highest incident rates of deaths, were the elderly within care home facilities. This group was high-risk for two reasons: they were more susceptible to the virus and could not cocoon as they require assistance/care. During the initial wave lasting around 5 months, approximately 600 deaths in 26 care home facilities occurred. To counter these issues, the Irish National Defense Forces personnel were deployed to caring/nursing homes during the second wave as two-thirds of nursing staff were unavailable to work (e.g., testing positive for COVID-19, living with vulnerable individuals). Initially, the National Defense Forces personnel also conducted the national contact tracing, later done by hired personnel. These young, energetic army personnel with the necessary skills/training caused a positive transformation in the wellbeing of the vulnerable elderly, by facilitating their daily activities and time outside (which was not previously possible due to staff shortages) for their mental health, which was deteriorating due to isolation.

Another significant challenge worldwide was the spreading of misinformed/ill-informed information, which affected the care given. For example, vaccine recipients regularly request vaccinations from specific producers (e.g., Pfizer, Moderna), scarcely seen with previous vaccination types. Furthermore, issues with mask-wearing and ventilation management caused confusion as the public was bombarded with often differing information. The sheer volume and intensity of media communication promoted a climate of fear, causing difficulties in public engagement.

For essential workers, occupational H&S guidelines were unavailable or too vague, and some recommendations came from unreliable sources. Many H&S professionals had to fill the gap without the required expertise, guidance and information, causing delayed deployment of appropriate controls.

As the pandemic progressed, it showed that speed often trumps perfection during dire events. The pandemic required rapid actions, even if there were shortcomings. The Irish government’s reluctance to utilise rapid antigen tests as they did not have the accuracy and specificity of polymerase chain reaction tests (Government of Ireland Press Statement) resulted in laboratory backlogs and slow response rate (i.e., ∼48 h up to 5 days).

The detachment of policymakers from frontline activities and the lack of synergies across all necessary stakeholders (e.g., H&S, engineering, medical professionals) still needs to be addressed. Earlier detection of issues could have occurred if the policymakers were engaging with essential workers. For example, the return of students to schools contributing to the spread of the virus was evident to teachers at a much earlier stage.

## Policy Options

### Policy Options and Recommendations

#### Protect the Most Vulnerable in Society

During the strictest restrictions in Ireland, people over seventy and medically vulnerable were advised to stay at home with limited social contacts (i.e., cocooning) and long-term residential care facilities were largely closed to visits. This resulted in extended isolation for many of the elderly community and caused a strain on nursing staff living with vulnerable people. With over 3,000 outbreaks and over 50,000 cases in nursing homes or community hospitals/long-stay units, including residential institutions (i.e., elderly, people with disabilities, hospices) and Irish healthcare workers were disproportionately affected by the COVID-19 pandemic [15]. A more robust pandemic preparedness plan for this cohort is essential, as 91% of COVID-19 deaths within the country were the elderly (persons aged 65 and over) and nearly a third in nursing homes (CSO). A strategy that alleviated many issues within these facilities was deploying personnel trained for disaster management (i.e., National Defense Forces), which should be considered earlier in future strategies. Future plans should also include the indirect effect of the restrictions and isolation on mental health among this group and younger and lower-risk individuals not being able to work/study and with limited social interactions.

#### Develop a Communication Strategy

Unprecedented in history was an international pandemic with a unique element: where digital technologies and social media platforms were used to share information, providing numerous opportunities and challenges. Universally, digital platforms shared vast amounts of information, including misleading information and extreme, polarising opinions. When limited information exists, it necessitates valuing any available data, and often, incorrect/irrelevant information contributes to a plethora of material, causing an “infodemic” [16]. Facilitating exchanges between policymakers and the general population is essential to encourage engagement and building trust through dialogue. Factors contributing to communication mistrust include delays in reporting, while a digital presence of unverified/misinformation is available. Though information provision must be balanced to prevent “pandemic fatigue” (i.e., perceived exhaustion to sustain the effort to respond to outbreaks), as people become accustomed to negative news, it becomes less impactful. Especially if personal COVID-19 effects were minor, they might perceive a lower risk and assess inaccurate/misleading information as correct [17]. Targeting communication where possible so as not to overwhelm people and to alleviate anxieties and uncertainty. Translating scientific findings with a more robust communication strategy would assist with engagement and addressing misinformation [2].

### Targeted Response

During the strictest lockdowns, essential services (e.g., emergency/medical care, essential supplies providers) are required to continue working. A review of the COVID-19 Prevention and Control Measures in Workplace Settings highlighted that identifying, understanding, and implementing effective workplace COVID-19 infection prevention and control (IPC) measures is critical to protect workers, their families, and communities. COVID-19 outbreaks and super-spreading events in these settings lead to changes in the community’s reproductive numbers [18]. A consensus in the scientific literature is that single measures are inadequate and require a multi-faceted approach to implementing controls. Comprehensive COVID-19 IPC measures include (but are not limited to) incorporating swift contact tracing and case isolation, personal protective equipment (PPE), and effectively preventing workplace outbreaks; relying on masking alone is not sufficient, nor is any single control measure [19]. Additionally, Irish essential workers wanted to access essential information early, specifically on local area events, going beyond reporting mere case numbers and more information on infection sources and clinical outcomes [17]. The infection risk at a given time has been shown to influence workers’ behaviour during a pandemic [7].

The risk of superspreader events could also be found among groups, for example, the Irish travelling community, a recognised minority group within Ireland, due to overcrowded living conditions. A pre-existing issue in Ireland was a housing and homelessness crisis for several years, allowing the virus to spread within emergency accommodations, typically overcrowded places, and similarly, asylum seekers in direct provision centres and people incarcerated in prisons. National pandemic preparedness plans must ensure an equitable response to encapsulate more vulnerable groups in society [20].

### Flexibility in Policy and Rapidly Learning From Other Nations

An Organization for Economic Cooperation and Development (OECD) report highlights that during events with catastrophic effects, it is essential to have real-time and rapid learning and sharing of lessons from different nations’ policies [2]. For example, during the pandemic, Ireland did not utilise rapid antigen tests, resulting in testing backlogs [Ireland had up to 40,000 people waiting up to 5 days for tests (Irish Times)] and reducing the frequency individuals could realistically test. Most nations used rapid tests for regular testing. The Minister of Health appointed the Expert Advisory Group on Rapid Testing (RTEAG) in July 2021, which concluded that Rapid Antigen Detection Tests (RADTs) should only be complementary to reverse transcription polymerase chain reaction (RT-PCR, commonly referred to as PCR) testing, as they demonstrated a lower overall detection sensitivity and test performance varied depending on administration (i.e., self-test, supervised test) (Interim Report).

An international example was the strong evidence that COVID-19 spreads by airborne transmission [21] and that workplace ventilation and non-pharmaceutical interventions needed improvement. Most public health authorities did not sufficiently address the transmission via air issue; more efficient and socially less disruptive exposure and risk reduction policies could have been implemented [22]. Particularly significant, as Ireland, after the first lockdown, stated facemasks were not mandatory, only on a government advisory basis. At this time, facemasks were recommended for other EU countries; though there was ambiguity in choosing appropriate masks and their availability, they were found to reduce the daily growth rate of infections by around 40% [23].

### Considering the Effects of the Control Measures Being Implemented

The aftermath of the pandemic has seen huge increases in mental health illnesses from the social stress of isolation and quarantine [5–8]. The consequences of forced isolation were worrying levels of loneliness, in particular affecting young adults due to social distancing measures [24]. Other mental health issues also emerged, including post-traumatic stress symptoms, confusion, and anger, and the stressors included quarantine duration, infection fears, frustration, boredom and inadequate supplies and information. To alleviate these adverse effects, quarantines should not last longer than necessary, and officials should provide a rationale for quarantine and protocols for the provision of sufficient supplies [8].

For workplaces that maintained operations, the need for mental health support included Employee Assistance Programmes with hybrid work schedules and reinforcement of control measures [5]. In particularly, healthcare workers should be extended these supports [12]. Customised interventions can potentially improve employee wellbeing during pandemics [5].

### Reflecting on Approaches Taken During the COVID-19 Pandemic

An international collective effort to tackle this crisis included the sharing of scientific information [1], the provision of open-access articles for freely accessible information, and companies sharing resources to better protect workers. Society played a crucial role in their adherence to national restrictions/lockdowns and high vaccination rates [13]. During the first COVID-19 vaccination programmes, Ireland had one of the highest vaccination uptakes worldwide. COVID-19 vaccinations have saved approximately 1.4 million lives in Europe and reduced mortality in Europe by 51% in the first 12 months [25].

The COVID-19 pandemic presented significant social, economic, and medical challenges and an economic cost. Workplace closures were reported as effective but economically costly [26], including job losses and the closure of all non-essential shops and services. Especially as Ireland’s first lockdown in 2020 was the longest in Europe, especially for hospitality and retail and wage subsidies and emergency COVID-19 Pandemic Unemployment Payment alone cost €9.2 billion [27].

With the end of the emergency phase of COVID-19, continuous caution due to surges in new virus variants, the short-lived nature of vaccine-induced immunity, sporadic reports of breakthrough infections, and sustained vaccine hesitancy pose considerable challenges for protecting the general population [28]. Hence, it is vital that we adopt the hierarchy of control methods to better protect public health [19].

### Preparing for the Next Pandemic Now

OECD’s first evaluations found pandemic preparedness was insufficient, as seen from the overwhelming loss of human life and economic costs [2]. Giménez, V. et al. conducted international comparisons of the COVID-19 response and found Ireland to have very high levels of effectiveness in pandemic management, though focused on the number of tests performed, positive tests and deaths [29]. A comparison with the inclusion of additional factors, such as the use of NPIs, disease severity, vaccination effectiveness and other risk factors (i.e., long COVID, mental health) across countries would be beneficial.

A pandemic preparedness plan requires three overarching aspects: preparedness, crisis management, and response and recovery [2]. More investment is needed in preparation for the next pandemic, including research and development [12], to have multi-faceted protection mechanisms comparable to the Swiss Cheese model [30], clear mandates and protocols for national shut-down procedures (i.e., what’s tolerable before restrictions are reinstated; the incident/date rate?, halting international flights) [12] and the recovery plan to include evaluating the impact of lockdowns (i.e., domestic violence, alcohol consumption, mental health) [2].

**FIGURE 3:**
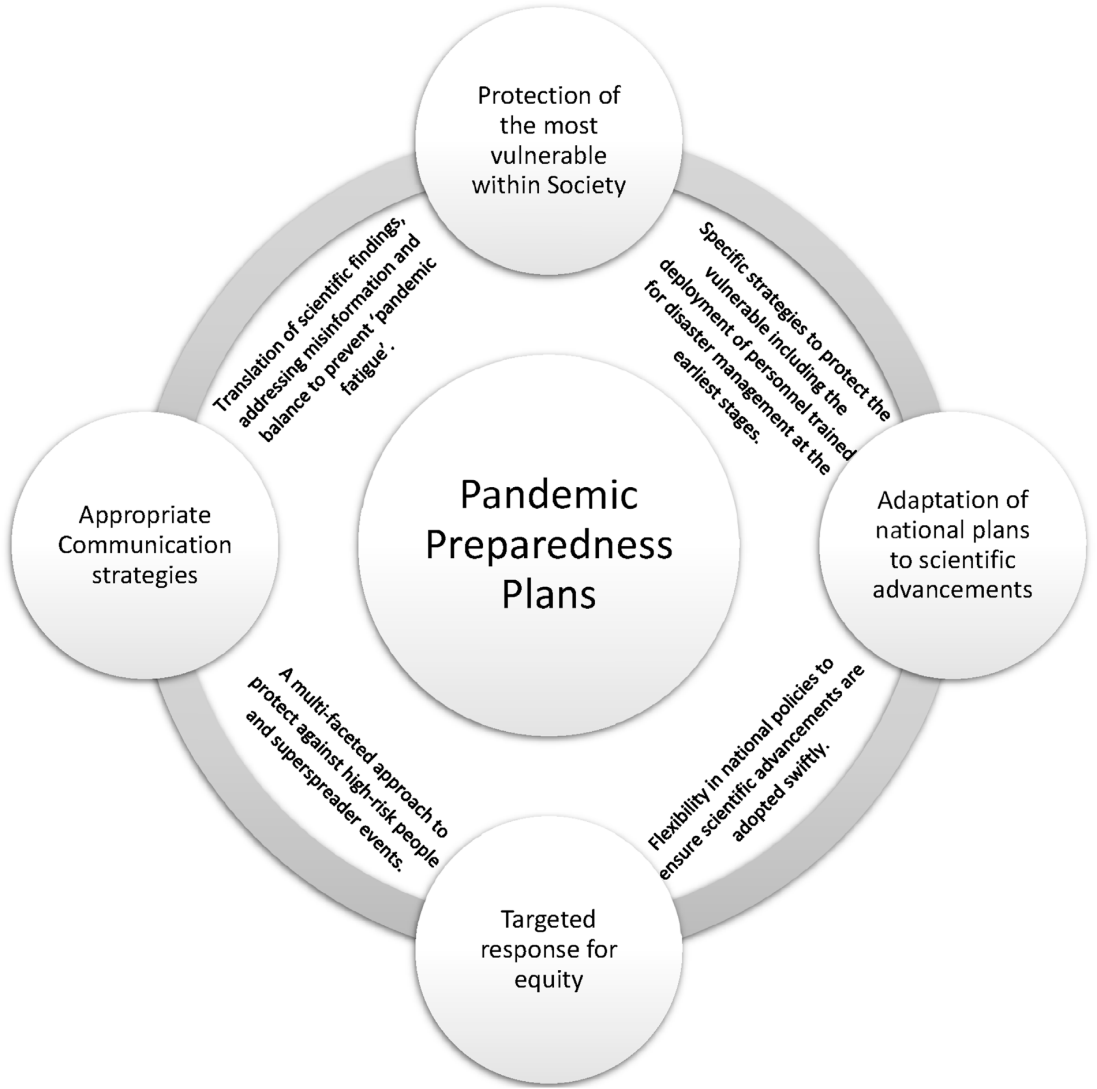
The areas identified during COVID-19 to address in the pandemic preparedness plans for future events (Ireland; 2022).

Lessons learnt include utilising our knowledge and resources swiftly and appropriately to achieve the most impact [12]. Governments must create a more holistic approach to health and wellbeing [31]. Synergies and close collaboration between multidisciplinary professions such as researchers, occupational H&S and public health professionals should be involved to a much higher degree [32, 33]. Some necessities in developing a more robust communication strategy include tackling ‘pandemic fatigue’ and two-way feedback mechanisms from the front line to policymakers to ensure they receive necessary information swiftly (Figure 3).

## Conclusion

The COVID-19 pandemic caused a worldwide disruption unparalleled to any in recent history. Ireland’s approaches and responses to the pandemic made it a unique experience.

Global collaboration is essential in preparing for future pandemics. The lessons learnt include ensuring that emerging scientific advancements are quickly reviewed and that policies/good practices for controlling the hazards, especially for vulnerable persons, are adopted. Our resources and personnel should be allocated where they can have the most impact and protect the most vulnerable.
